# Supplementary material for: Empirical methods for controlling false positives and estimating confidence in ChIP-Seq peaks
Source: BMC Bioinformatics. 2008 Dec 5;9:523. doi: 10.1186/1471-2105-9-523 (PMC2628906; doi:10.1186/1471-2105-9-523)
Supplement: Additional File 1 — A variety of html documents from the USeq web site detailing the available applications, their best usage, output file type descriptions, command line menus, etc. [file 1471-2105-9-523-S1.zip › USeqUserGuides/JavaDoc.zip!/JavaDoc/util/bio/parsers/gff/package-summary.html]

util.bio.parsers.gff


|  |  |  |
| --- | --- | --- |
| **Overview** \| **Package** \| Class \| **Use** \| **Tree** \| **Deprecated** \| **Index** \| **Help** | |  |
| **PREV PACKAGE**   **NEXT PACKAGE** | **FRAMES**    **NO FRAMES**     **All Classes** |  |


---

## Package util.bio.parsers.gff

GFF2 and GFF3 parsers.

**See:**
  
          **Description**

| **Class Summary** | |
| --- | --- |
| **DmelRel4Extractor** | Class for building gene models from GFF3Features, specifically tailored to release 4.0 drosophila melanogaster GFF. |
| **ExtractGffFeatures** |  |
| **GadFlyGffExtractor** | Class for building annotation objects from GffFeatures, basically a converter, specific to the old dmel GadFly annotation. |
| **Gff3Feature** | A container for each of the values in a GFF3 line. |
| **Gff3Parser** | For parsing a GFF3 text file into GFF3Feature objects, be sure to set the regExTypes String filter to match which types you want to save! |
| **GffFeature** | A container for each of the values in a GFF 2 line. |
| **GffParser** | Parses GFF files extracting the annotation according to GFF(2). |
| **ParseWormBaseGffToUCSC** |  |

## Package util.bio.parsers.gff Description

GFF2 and GFF3 parsers.

---


|  |  |  |
| --- | --- | --- |
| **Overview** \| **Package** \| Class \| **Use** \| **Tree** \| **Deprecated** \| **Index** \| **Help** | |  |
| **PREV PACKAGE**   **NEXT PACKAGE** | **FRAMES**    **NO FRAMES**     **All Classes** |  |


---
